# Viperin Inhibits Enterovirus A71 Replication by Interacting with Viral 2C Protein

**DOI:** 10.3390/v11010013

**Published:** 2018-12-26

**Authors:** Chunyu Wei, Caishang Zheng, Jianhong Sun, Dan Luo, Yan Tang, Yuan Zhang, Xianliang Ke, Yan Liu, Zhenhua Zheng, Hanzhong Wang

**Affiliations:** CAS Key Laboratory of Special Pathogens and Biosafety, Center for Emerging Infectious Diseases, Wuhan Institute of Virology, Chinese Academy of Sciences, Wuhan 430071, China; weichunyu08@outlook.com (C.W.); z-cshang@163.com (C.Z.); sunjianhong0604@sina.com (J.S.); luodan64@126.com (D.L.); tangyan_0209@163.com (Y.T.); zhangyuan@wh.iov.cn (Y.Z.); xianke909@163.com (X.K.); liuy@wh.iov.cn (Y.L.); wanghz@wh.iov.cn (H.W.)

**Keywords:** Enterovirus A71, interferon-stimulated gene, viperin, 2C protein, virus-host interaction

## Abstract

Enterovirus A71 (EVA71) is a human enterovirus belonging to the Picornaviridae family and mostly causes hand-foot-and-mouth disease in infants. Viperin is an important interferon-stimulated gene with a broad antiviral activity against various viruses. However, the effect of viperin on human enteroviruses and the interaction mechanism between EVA71 and viperin remains elusive. Here, we confirmed the EVA71-induced expression of viperin in a mouse model and cell lines and showed that viperin upregulation by EVA71 infection occurred on both the mRNA and protein level. Viperin knockdown and overexpression in EVA71-infected cells indicated that this protein can markedly inhibit EVA71 infection. Interestingly, immunofluorescent confocal microscopy and co-immunoprecipitation assays indicated that viperin interacts and colocalizes with the EVA71 protein 2C in the endoplasmic reticulum. Furthermore, amino acids 50–60 in the N-terminal domain of viperin were the key residues responsible for viperin interaction with 2C. More importantly, the N-terminal domain of viperin was found responsible for inhibiting EVA71 replication. Our findings can potentially aid future research on the prevention and treatment of nervous system damage caused by EVA71 and may provide a potential target for antiviral therapy.

## 1. Introduction

The antiviral response of the innate immune system is mainly mediated by the activated expression of interferon stimulated gene (ISG) [1]. Hundreds of ISGs have been identified since their discovery more than 25 years ago, but only a few have been characterized based on their antiviral activity [2]. For instance, IFITM proteins, including IFITM1, IFITM2, and IFITM3, potently or at least partially inhibit human immunodeficiency virus replication by interfering with viral entry [3,4]. Viperin (virus inhibitory protein, endoplasmic reticulum [ER]-associated, IFN-inducible) is one of the few ISGs proven to have direct antiviral activity and can inhibit the replication of multiple viruses, attracting increasing research attention [5].

Enterovirus A71 (EVA71) is a human enterovirus belonging to the Picornaviridae family and is one of the main pathogens of hand-foot-and-mouth disease, which has had more than 10 outbreaks worldwide since it was first reported in 1969 [6]. EVA71 has become one of the most neurotoxic enteroviruses after the elimination of poliovirus. The RNA genome of EVA71 contains approximately 7400 nucleotides. After a virus enters the host cell, viral RNA is directly translated into a polyprotein, after which virus-specific proteases cleave the polyprotein into structural and nonstructural proteins. This process produces 10 mature viral proteins and other intermediates that exercise several specific functions in the virus life cycle [7]. 2C, which is encoded by the P2 region of viral genomic RNA, is a nonstructural protein that plays an important role in the virus life cycle [6]. 2C functions as both an RNA helicase and ATP-independent RNA chaperone in EVA71 infection [8] and affects enterovirus morphogenesis by direct interaction with VP3, a capsid protein [9]. 2C also binds to host proteins Reticulon-3 and inhibitor kappa B kinaseβ (IKKβ) during viral infection to facilitate viral replication and antiviral immunity escape [10,11].

Viperin is an ISG that is highly conserved from lower vertebrates to mammals. It is structurally divided into an N-terminal double alpha helical domain, intermediate SAM region, and the C-terminal domain. The double alpha helical region localizes viperin to the ER and cell membrane lipid raft structure, while viperin antiviral activity is associated with the C-terminal domain. The antiviral area in the C-terminus is highly conserved and is the key domain structure that suppresses replication of hepatitis C virus (HCV) and dengue virus (DENV) [12]. An increasing number of studies have indicated that viperin exhibits broad antiviral activity in vitro and can inhibit both RNA and DNA viral replication.

Studies have shown that viperin suppresses the replication of influenza A and HIV-1 by blocking the release of viral particles [13,14]. Moreover, viperin inhibits the replication of members of the Flaviviridae, such as HCV, DENV. Viperin interacts with the RC of HCV and DENV to inhibit viral replication by binding with the nonstructural HCV protein NS5A and the DENV NS3 protein [15,16]. Other studies have also shown that viperin interacts with the associated protein A of vesicle-related membrane protein (VAP-A) of the HCV RC and inhibits viral replication [16]. Viperin overexpression in HCMV-infected human fibroblasts remarkably reduced expression of the structural proteins indispensable for viral assembly and maturation [17]. Meanwhile, HCMV-induced viperin disrupts cellular metabolism to enhance the infectious process by localization from the ER to the mitochondria [18]. Viperin was also shown to inhibit replication of other viruses in vitro, such as Chikungunya virus and Sindbis virus [19,20,21,22]. Importantly, viperin was reported to inhibit both replication and assembly of TBEV via targeting the viral NS3 protein and the cellular protein Golgi brefeldin A-resistant guanine nucleotide exchange factor 1 (GBF1) respectively [23,24]. Moreover, viperin catalyzes cytidine triphosphate (CTP) to 3ʹ-deoxy-3′,4ʹ-didehydro-CTP (ddhCTP), which acts as a chain terminator for the RNA-dependent RNA polymerases of flavivirus including ZIKA virus and West Nile Virus [25]. However, the mechanism behind the replication inhibition of other RNA viruses by viperin remains unclear.

In this study, we showed that viperin mRNA is upregulated in EVA71-infected human astrocytoma cells. Furthermore, we aimed to confirm the effects of EVA71 infection on viperin expression and to investigate the capacity of viperin in functioning as an antiviral protein against EVA71. Moreover, we attempted to verify the interaction between EVA71 and viperin in cells and found that EVA71 upregulated viperin expression in both the mouse and cell infection model. Viperin also interacted with the 2C protein of EVA71 and inhibited viral replication through its N-terminal domain. Therefore, our findings provide insights into the pathogenesis of EVA71 neurological complications and offer a potential target for antiviral therapy

## 2. Materials and Methods

### 2.1. Virus, Cell Culture, Transfection, and Infection

Human embryonic kidney 293T cells (293T; China Center for Type Culture Collection, Wuhan, China) and human rhabdomyosarcoma (RD) cells (ATCC-CCL-136) were cultured in Dulbecco’s modified Eagle’s medium (Life Technologies, Carlsbad, CA, USA). Human neuroblastoma cells SK-N-SH cells (ATCC-HTB-11) were cultured in minimum essential medium (Life Technologies, Carlsbad, CA, USA). Human astrocytoma CCF-STTG1 cells (ATCC-CRL-1718) were cultured in RPMI 1640 (Life Technologies, Carlsbad, CA, USA). All cells were maintained in medium containing 10% fetal bovine serum (Gibco, Rockville, MD, USA) and incubated at 5% CO_2_ and 37 °C.

Cells were plated in six-well plates overnight and transfected with 2 µg of plasmid per well or siRNA with the Calcium Phosphate-mediated ProFection Mammalian Transfection System (Promega, Madison, WI, USA) or Lipofectamine™ 2000 reagent (Invitrogen, Carlsbad, CA, USA) according to manufacturer’s instructions. CCF-STTG1 cells and 293T cells were plated in six-well plates overnight and infected with EVA71 (BrCr strain) for the indicated time points.

### 2.2. Antibodies and Sirna

The main antibodies used were anti-EVA71 VP1 rabbit poly-antibody (produced in-house), rabbit anti-HA (Sigma-Aldrich, St. Louis, MO, USA), mouse anti-FLAG (Sigma-Aldrich, St. Louis, MO, USA), and rabbit anti-viperin (Cell Signaling Technology, Danvers, MA, USA). Three siRNA sequences targeting the viperin transcript (Si-viperin-1, -2, and -3) and negative control siRNA were designed and synthesized (Table 1) were ordered from RiboBio (Guangzhou, China). For verification of knock down efficiency of the siRNAs, the siRNAs were transfected to 293T cells. At 24 h post-transfection, cells were treated with type I interferon (1000 U/mL) for 6 h and subsequently lysed to detect viperin protein in the cells using western blot.

### 2.3. Plasmids

FLAG-viperin and GFP-viperin were constructed by inserting the PCR products obtained using the primers described in Table 1 into the plasmids pCAGGS and pEGFP-N1, respectively. 2C-HA and were constructed previously [11]. The viperin mutants FLAG-N-viperin, FLAG-C-viperin, FLAG-ΔSAM (1 + 2, 2 + 3, and 3 + 4), FLAG-ΔSAM (1 + 2, 2 + 3, and 3 + 4), FLAG-ΔN, FLAG-ΔSAM, FLAG-ΔC, FLAG-ΔN-42aa, FLAG-ΔN-50aa, FLAG-ΔN-60aa, and FLAG-ΔN-65aa were constructed using multistep overlap PCR amplification with the primers described in Table 1, and subsequently inserted into the expression vector pCAGGS as previously described [11] through *Xho*I and *Not*I restriction enzyme digestion.

### 2.4. Animal Models

Two-week-old BALB/c mice were purchased from HuBei Center for Disease Control (CDC) (Wuhan, China) and were infected with 100 µL of high-virulence EVA71 (GZ-CII strain) through an intraperitoneal injection at a dose of 10^5^ TCID_50_/mouse. Subsequently, mice body weight and symptoms were recorded every day. The mice were sacrificed after they displayed oblivious posterior paralysis. Brain tissues of mice infected with EVA71 or injected with control PBS buffer were obtained for immunohistochemistry and RNA isolation.

All studies were conducted in strict accordance with the institutional guidelines for animal research and approved by the Administration of Affairs Concerning Experimental Animals of the People’s Republic of China. All mice were housed under specific-pathogen-free conditions in individually ventilated caging systems. All animal experiments were reviewed and approved in advance by the Ethics Committee of the Animal House Facility at the Wuhan Institute of Virology, Chinese Academy of Sciences (Wuhan, China; protocol number, WIVA07201705; approval date, 10 May 2017).

### 2.5. Immunohistochemistry

The brain tissues of mice were embedded in paraffin and cut into 5-μm sections. After deparaffinization and rehydration, the sections were incubated in Tris-buffered saline (TBS) containing 0.1 Triton X-100 for 20 min at room temperature and then washed for 10 min in TBS. To eliminate endogenous peroxidase, the sections were treated with 2% H_2_O_2_ in methanol for 20 min. The sections were then blocked in TBS containing 10% normal rabbit serum (vector Lab, Burlingame, CA, USA) and 0.2% bovine serum albumin (BSA) for 30 min with shaking at room temperature. Subsequently, brain sections were incubated with a mouse anti-EVA71 VP1 antibody and rabbit anti-viperin antibody (1:200) overnight at 4 °C. The sections were washed in TBS and incubated with Texas Red-labeled and fluorescein isothiocyanate-labeled secondary antibodies (1:500; Invitrogen) for 1 h at room temperature. The sections were washed in TBS five times, counterstained with DAPI (Sigma-Aldrich, St. Louis, MO, USA) for 8 min at room temperature, washed in TBS, and then embedded in Mowiol (Sigma-Aldrich, St. Louis, MO, USA). Slides were cover-slipped with Vectashield Mounting Medium (Vector Lab, Burlingame, CA, USA) and imaged under a fluorescent microscope.

### 2.6. Co-IP and Immunoblotting Assays

After 293T cells were transfected with expression plasmids or vectors for 24 h, the cells were harvested and lysed with western and IP lysis buffer containing the protease inhibitor phenylmethylsulfonyl fluoride. The cells were then centrifuged at 12,000× *g* for 15 min at 4 °C and the supernatants collected in 1.5 mL tubes. After adding 2 µL anti-FLAG antibodies to the supernatants, the tubes were gently shaken overnight at 4 °C. Approximately 20 µL of Protein G Agarose beads (GE Healthcare, Diegem, Belgium) washed five times with PBS were then added to the tubes and gently shaken at room temperature for 1 h. The beads were washed with 500 µL 0.02% PBST (1× PBS + 0.02% Triton 100) five times before adding 100 µL 2× sodium dodecyl sulfate (SDS) loading buffer and boiling for 10 min to separate immunoprecipitates from the beads. The immunoprecipitates were then subjected to SDS polyacrylamide gel electrophoresis and western blotting.

### 2.7. RNA Extraction and Reverse Transcription–Quantitative PCR (RT-qPCR) Analysis

Total RNA was extracted using TRIzol reagent (Invitrogen, Carlsbad, CA, USA) according to manufacturer’s instructions. RT-PCR was performed using 1 µg RNA with a random primer and reverse transcriptase (Takara Bio, Shiga, Japan). Quantitative PCR was performed using SYBR Green Master Mix (Bio-Rad, Hercules, CA, USA) and the CFX96 Touch Real-Time PCR Detection System (Bio-Rad). Each reaction ran in triplicate following the protocol of our previous study [26]. Calibration curves were generated using plasmids carrying target genes. the square of related coefficient (R^2^) of the standard curve >0.99, and the amplification efficiency ranging between 95% and 110%. GAPDH was used as normalization control for obtaining the relative expression levels of VP1. The primers used are indicated in Table 1.

### 2.8. Immunofluorescence and Confocal Microscopy

293T cells were transfected with GFP-viperin and HA-2C plasmids using a Lipofectamine™ 2000 reagent according to manufacturer’s instructions. After 24 h, cells were washed with 3% normal goat serum (NGS) in PBS (all subsequent washing steps were performed with 3% NGS in PBS), fixed with 4% paraformaldehyde for 15 min, washed, permeabilized with 0.2% Triton X-100 in PBS for 15 min, washed, blocked with 5% NGS and 2% BSA in PBS for 1 h, and then washed three times. For immunostaining of 2C-HA, cells were incubated with rabbit anti-HA antibody overnight at 4 °C. After five washes (10 min each), the cells were incubated with TRITC-conjugated goat anti-rabbit IgG (Jackson Immune Research, West Grove, PA, USA) for 1 h at room temperature. After five washes (10 min each), cell nuclei were stained with Hoechst 33258 (Beyotime Institute of Biotechnology, Nanjing, China) and washed three times (5 min each). For ER staining, cells were fixed with 4% paraformaldehyde and then ER-Tracker Blue-White DPX (ThermoFisher Scientific, Grand Island, NY, USA) were applied before fixation. Fluorescent images were obtained using a Perkin Elmer UltraView VOX confocal microscope equipped with 405 nm (for violet fluorescence of the stained cell nuclei), 488 nm (green fluorescence), and 561 nm (red fluorescence) excitation lasers. The cells were imaged with a 60× oil immersion objective.

### 2.9. Statistical Analysis

All experiments were repeated and conducted in triplicate. Data are represented as the mean ± standard deviation (SD) where indicated and used for all statistical analyses with GraphPad Prism 6.0 software (GraphPad Software Inc., La Jolla, CA, USA). Differences were considered statistically significant when *p* < 0.05.

## 3. Results

### 3.1. EVA71 Induced Viperin Expression In Vitro and In Vivo

**Figure 1:**
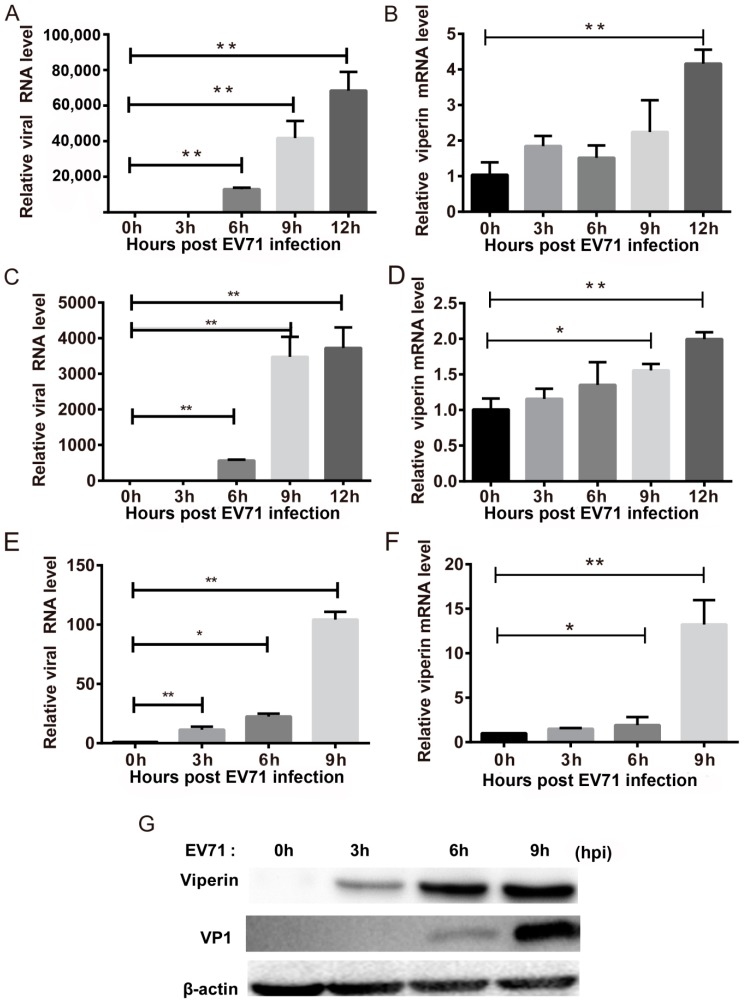
EVA71 induces viperin expression in vitro. (**A**,**B**) RD, (**C**,**D**) SK-N-SH, and (**E**,**F**) CCF-STTG1 cells were infected with EVA71 at a multiplicity of infection (MOI) = 1 for the indicated time points. The relative expression of viperin was detected using quantitative polymerase chain reaction (q-PCR) using glyceraldehyde 3-phosphate dehydrogenase (GAPDH) as the control. After the cells were infected with EVA71 at an MOI = 1, levels of viperin mRNA and viral RNA of EVA71 were detected using real-time q-PCR at the indicated time points. Data are representative of at least three independent experiments performed in triplicate (mean ± SD of fold change). * *p* < 0.05 and ** *p* < 0.01. (**G**) Western blot analysis of viperin protein, VP1 protein, and β-actin of EVA71-infected CCF-STTG1 cells at the indicated time points.

To confirm the effects of EVA71 on viperin expression, we analyzed viperin expression in EVA71-infected cell lines and a mouse model. In EVA71-infected RD cells, the viral RNA of EVA71 was found to be significantly increased at each indicated time point post-infection (Figure 1A), while viperin mRNA (Figure 1B) was upregulated only at 12 h post-infection (hpi) but not increased significantly at other time points. In the human neuroblastoma cell line SK-N-SH, EVA71 RNA levels were also elevated at each indicated time point (Figure 1C) and viperin mRNA levels were found increased at 9 hpi and 12 hpi (Figure 1D). Moreover, we determined viperin expression in another human neuron cell line, CCF-STTG1; as shown in Figure 1E,F, q-PCR revealed that viperin mRNA levels increased significantly at 6 hpi and 9 hpi (Figure 1F) and that viral RNA levels were also increased significantly at each indicated time point (Figure 1E). Furthermore, western blotting showed that levels of both viperin and the VP1 protein of EVA71 were markedly increased (Figure 1G). Collectively, all the three types of cell including the neurogenic cell (SH-N-Sk and CCF-STTG1) and non-neurogenic cells (RD cell) could be infected by EV71 and could express viperin during EVA71 infection. However, both of the neurogenic cells were more sensitive in induction of viperin at early stage of infection.

**Figure 2:**
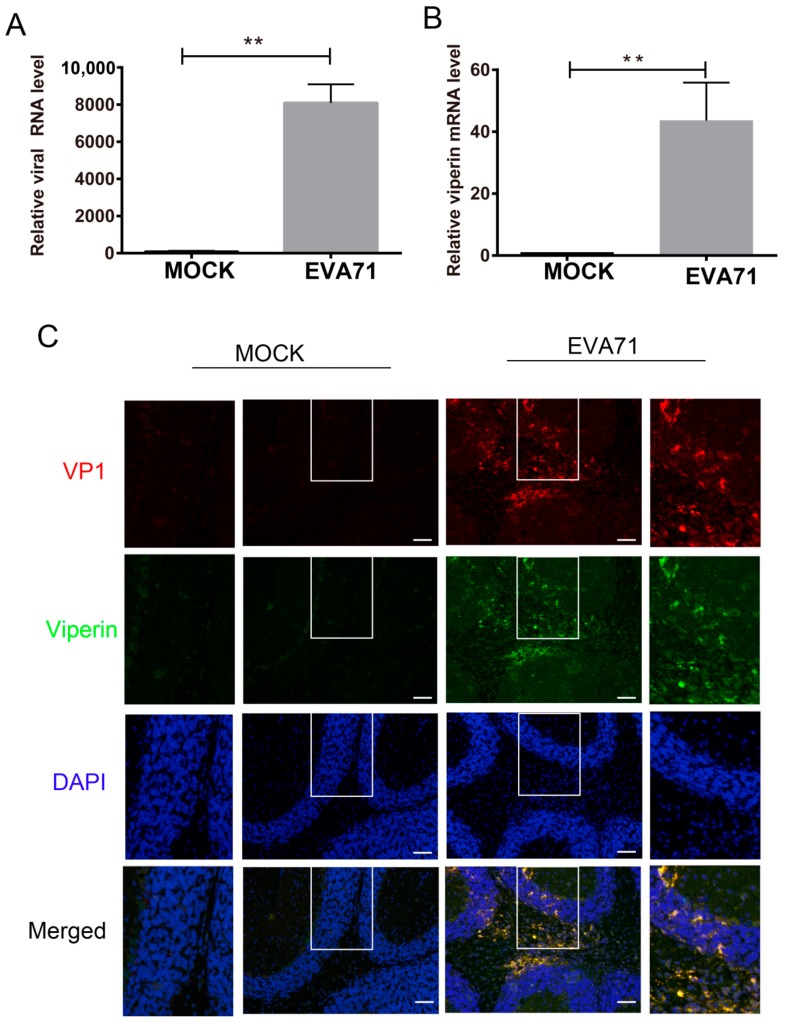
EVA71 induced viperin expression in vivo. Two-week-old BALB/c mice were infected with high-virulence EVA71 (GZ-CII strain), and the relative expression levels of (**A**) viperin and (**B**) EVA71 viral RNA in brain tissues were measured using q-PCR with GAPDH as a control. Data are representative of at least three independent experiments performed in triplicate (mean ± SD of fold change). ** *p* < 0.01. (**C**) Double-labeling immunohistochemistry analysis of EVA71 VP1 and viperin in the brain tissue was performed. Green fluorescence represents viperin protein, red fluorescence represents EVA71 VP1 protein, and blue fluorescence represents nuclei. The scale bar in the images is 20 μm. The enlarged images corresponding to the rectangle areas are shown next to the original image.

In parallel, we also utilized the EVA71 mouse infection model to detect viperin expression. Two-week-old BALB/c mice were infected via intraperitoneal injection of a high-virulence EVA71 (GZ-CII strain) [27]. After 4 days, the mice were sacrificed when they showed oblivious posterior paralysis. As the brain tissue is the most sensitive tissue for high-virulence EVA71 infection, the levels of viperin mRNA and EVA71 viral RNA in brain tissues were detected using q-PCR. Compared with the mock-treated group, viperin expression in EVA71-infected mice increased significantly by approximately 40-fold (Figure 2A). Thus, 1.08 × 10^4^ copies of EVA71 genomic RNA were detected in per mg total RNA extracted for the EV71 infected mouse brain (Figure 2B). Additionally, immunofluorescence assays showed that viperin protein co-existed with EV71 VP1 protein in most cells within the hippocampus of the brain (Figure 2C). This result indicated that EV71 infection stimulated viperin expression in mouse brain tissue.

### 3.2. Viperin Inhibited EVA71 Replication

**Figure 3:**
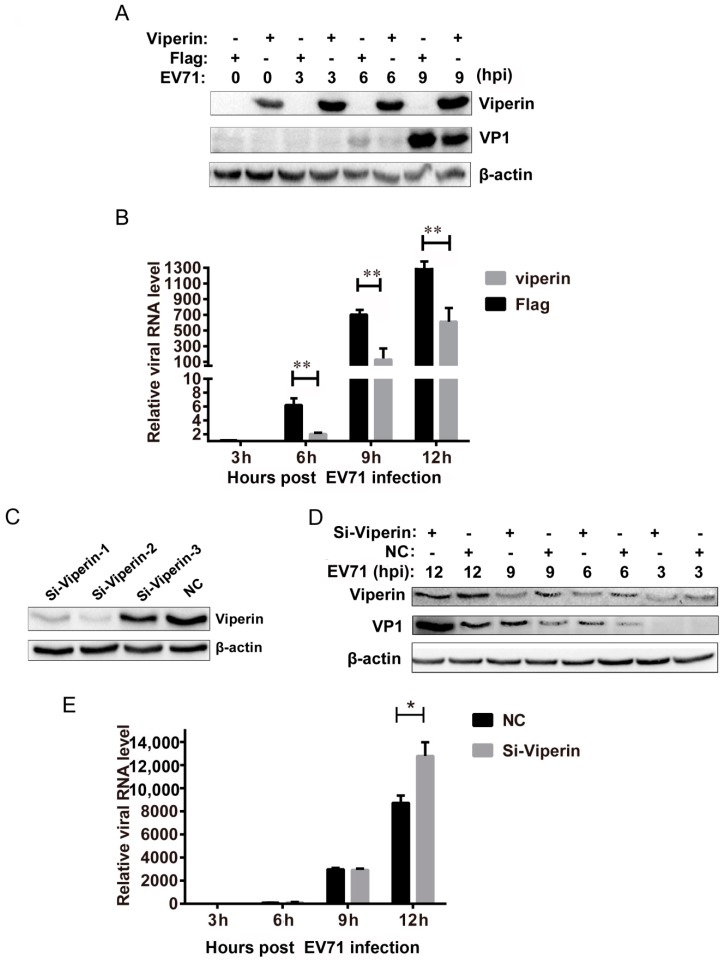
Viperin suppresses EVA71 replication. 293T cells were transfected with FLAG or FLAG-viperin for 24 h and then infected with EVA71 at an MOI = 1 for the indicated times. Subsequently, expression levels of VP1 were detected by (**A**) western blotting and (**B**) q-PCR with GAPDH as a control. (**C**) Synthesized siRNAs—Si-viprin-1, Si-viprin-2, and Si-viprin-3—or negative control (NC) were used to transfected 293T cells. At 24 h post-transfection, cells were treated with type I interferon for 6 h and lysed to determine siRNA efficiency by detecting viperin protein through western blotting. (**D**) 293T cells were then transfected with viperin siRNA or negative control for 24 h followed by infection with EVA71 at an MOI = 1 for the indicated times. Expression levels of VP1 were measured using western blotting. (**E**) q-PCR analysis with GAPDH as control to detect the viral RNA of EVA71 of viperin siRNA transfected 293T cells at indicated time points. Data are representative of at least three independent experiments performed in triplicate (mean ± SD of fold change). * *p* < 0.05 and ** *p* < 0.01.

To determine the effects of viperin on EVA71 infection, viperin was overexpressed and knocked down during EVA71 infection. 293T cells were transfected with a FLAG-viperin expression plasmid or a control empty plasmid. At 24 h post-transfection, cells were infected with EVA71. As shown in Figure 3A, Western blot analysis indicated that the EVA71 VP1 protein was also downregulated at indicated EVA71 post-infection time points. q-PCR also indicated that EVA71 viral RNA levels decreased at various time points post-infection (6, 9, and 12 hpi) (Figure 3B). These results indicate that viperin overexpression significantly suppressed EVA71 infection. Additionally, viperin was knocked down by transfecting EVA71-infected cells with siRNA. Three siRNA sequences were used to transfect 293T cells, while nonspecific RNA was used as a control. At 24 h post-transfection, cells were treated with type I interferon for 6 h and subsequently lysed to detect viperin protein in the cells; as shown in Figure 3C, Si-viperin-2 was the most efficient siRNA and was subsequently chosen for knockdown experiments. Thus, 293T cells were first transfected with siRNA followed via infection with EVA71 at a multiplicity of infection (MOI) = 1 at 24 h post-transfection. As shown in Figure 3D, knockdown with Si-viperin enhanced protein levels of EVA71 VP1 at 6, 9, and 12 hpi compared with the negative control. Moreover, q-PCR analysis indicated that Si-viperin transfection also increased EVA71 viral RNA levels at the indicated post-infection time points (Figure 3E). These findings indicate that viperin knockdown facilitated EVA71 infection. Collectively, the results of viperin overexpression and knockdown in EVA71-infected cells demonstrated that viperin can inhibit EVA71 replication in host cells.

### 3.3. Viperin Was Colocalized with EVA71 2C in the ER

**Figure 4:**
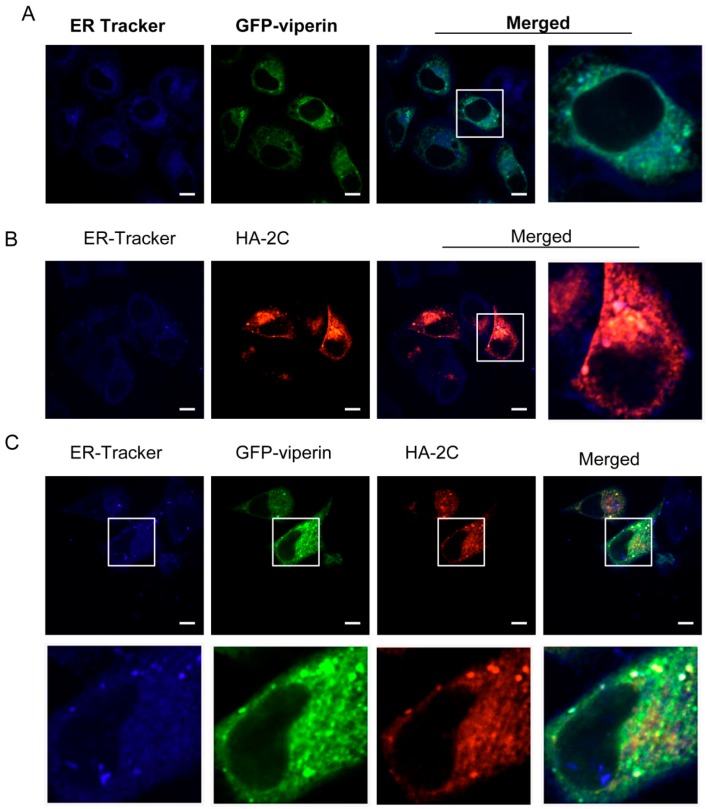
Viperin colocalization with EVA71 2C in the endoplasmic reticulum (ER). 293T cells were transfected with (**A**) GFP-viperin, (**B**) HA-2C, or (**C**) co-transfected with HA-2C and GFP-viperin for 24 h. The cells were fixed and stained with ER-Tracker Blue-White for ER labeling and then visualized under a fluorescent microscope. In the above experiments, viperin (green) and HA-2C (red) are shown, as well as ER (blue) counterstained with ER-Tracker. The enlarged images corresponding to the rectangle areas are shown next to (A and B) or below (C). Scale bar is 10 μm.

Viperin is localized in and is associated with the ER [28]. In addition, the 2C protein of enteroviruses is reported to be associated with the intracellular membrane network [29]. Thus, we investigated whether the 2C protein of EVA71 is associated with viperin in the ER. GFP-viperin and HA-2C plasmids were used to transfect 293T cells for 24 h and ER-Tracker Blue-White was used to label the ER. Through immunofluorescence, we analyzed the localization of viperin and 2C and as shown in Figure 4A, viperin was found mostly localized to the ER. The 2C protein was also found localized to the ER (Figure 4B). Furthermore, we co-transfected 293T cells with GFP-viperin and HA-2C plasmids for 24 h. Immunofluorescent confocal microscopy showed that most viperin-GFP were colocalized with HA-2C in the ER (Figure 4C), suggesting that 2C and viperin may have interacted in the ER.

### 3.4. EVA71 2C Interacted with Viperin

**Figure 5:**
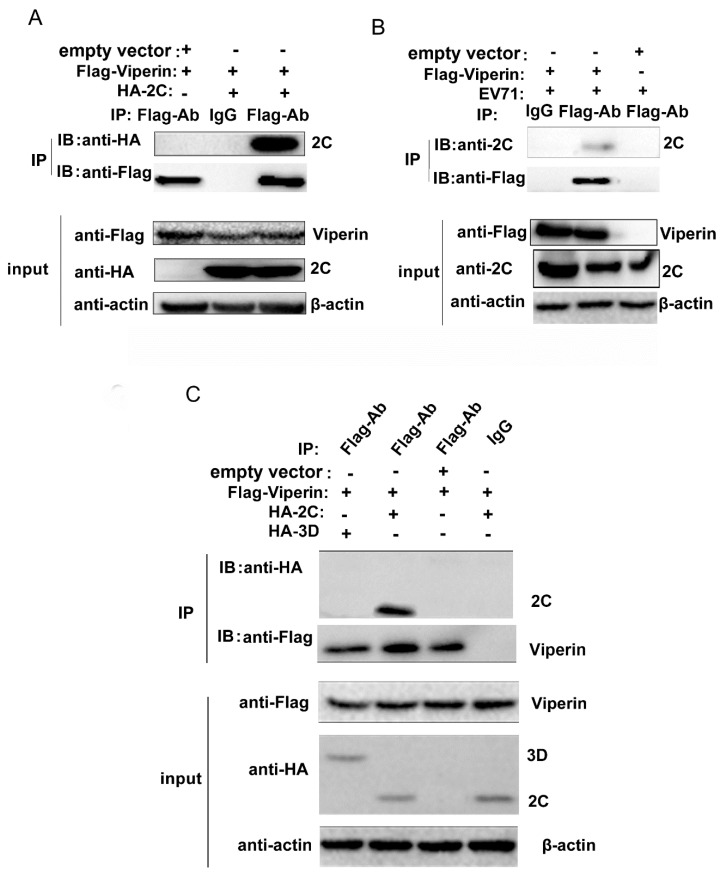
EVA71 2C interacted with viperin. FLAG-viperin was (**A**) co-transfected with HA-2C into 293T cells or (**B**) transfected alone into 293T cells that were then infected with EVA71 at an MOI = 1. After 24 h, the cells were lysed and analyzed by co-immunoprecipitation (co-IP) and immunoblotting assays. (**C**) HA-2C, HA-3D, and the empty expressing vector pCAGGS-HA were co-transfected with FLAG-viperin into 293T cells for 24 h, and the cells were lysed and analyzed by co-immunoprecipitation (co-IP) and immunoblotting assays with indicated antibodies.

To verify whether EVA71 2C binds to viperin, we utilized a co-IP assay to confirm the interaction between 2C and viperin. FLAG-viperin and HA-2C plasmids were used to co-transfect 293T cells and then the cells were lysed 24 h post-transfection. HA-2C was detected in the precipitate with the anti-FLAG antibody (Figure 5A). Furthermore, the interaction between viral 2C and viperin was analyzed during EVA71 infection after transfecting 293T cells with FLAG-viperin. At 24 h post transfection, the cells were infected with EVA71 at an MOI = 1 and then lysed at 12 h post infection. The 2C protein was detected in the precipitate with the anti-FLAG antibody (Figure 5B). To further confirm whether 3D protein, another viral protein that is critical for virus replication, interacts with viperin, HA-2C and HA-3D were transfected with FLAG-viperin into 293T cells respectively for co-IP assay. As shown in Fig 5C, although the HA-2C was detected in the precipitate with the anti-FLAG antibody, HA-3D was not detected in the precipitate. This result indicates that 3D could not interact with viperin and it suggests that the interaction between 2C and viperin was specific.

### 3.5. The N-Terminal Domain Was Responsible for Viperin Binding to 2C

**Figure 6:**
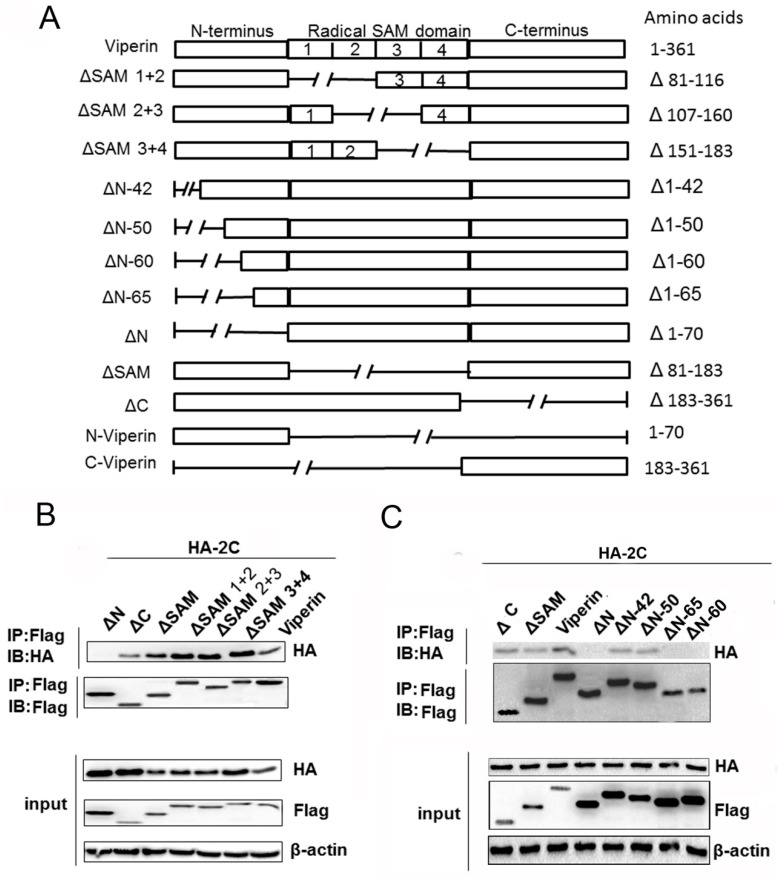
The N-terminal domain is responsible for viperin binding to 2C. (**A**) Schematic diagram of a series of viperin deletion mutants. (**B**) FLAG-viperin, ΔSAM3+4, ΔSAM2+3, ΔSAM1+2, ΔSAM, ΔC, or ΔN were co-transfected with HA-2C into 293T cells for 24 h. The cells were then lysed, followed by co-IP and immunoblotting assays. (**C**) 293T cells were co-transfected HA-2C with ΔC, ΔSAM, viperin, ΔN, ΔN-42, ΔN-50, ΔN-65, or ΔN-50 for 24 h, and then the cells were lysed and used for co-IP and immunoblotting.

To identify the viperin domain responsible for interacting with 2C, a series of mutants was constructed based on the functional domains of viperin. As illustrated in Figure 6A, viperin mutants ΔSAM (1 + 2), ΔSAM (2 + 3), ΔSAM (3 + 4), ΔN, ΔSAM, ΔC, ΔN-42aa, ΔN-50aa, ΔN-60aa, and ΔN-65aa were constructed. The domain responsible for viperin binding to 2C was initially analyzed using co-IP. The FLAG-tagged mutants, ΔSAM (1 + 2), ΔSAM (2 + 3), ΔSAM (3 + 4), ΔN, ΔSAM, and ΔC were co-transfected with HA-2C plasmids into 293T cells for 24 h. The anti-HA antibody was used to detect 2C protein immunoprecipitated with the anti-FLAG antibody. All viperin mutants were able to bind to 2C except for the ΔN mutant (Figure 6B). Thus, the N-terminal domain of viperin may be the key domain responsible for binding with 2C.

To determine the amino acids in the N-terminal domain responsible for binding to 2C, we analyzed interactions between more viperin mutants and 2C using co-IP. The FLAG-tagged viperin, viperin mutants (ΔN-42aa, ΔN-50aa, ΔN-60aa, ΔN-65aa, and ΔN), and HA-2C plasmids were co-transfected into 293T cells. At 24 h post-transfection, the cells were lysed and an anti-HA antibody was used to detect the 2C protein immunoprecipitated with the anti-FLAG antibody. Surprisingly, we found that ΔN-60aa, ΔN-65aa, and ΔN did not interact with 2C, whereas other mutants, including ΔN-50aa, could bind to 2C, similarly to the full-length viperin (Figure 6C). Therefore, the results suggest that the 50–60 aa region in the viperin N-terminal domain was required for interaction with 2C.

### 3.6. The N-Terminal Domain Was Responsible for Viperin Inhibition of EVA71 Replication

**Figure 7:**
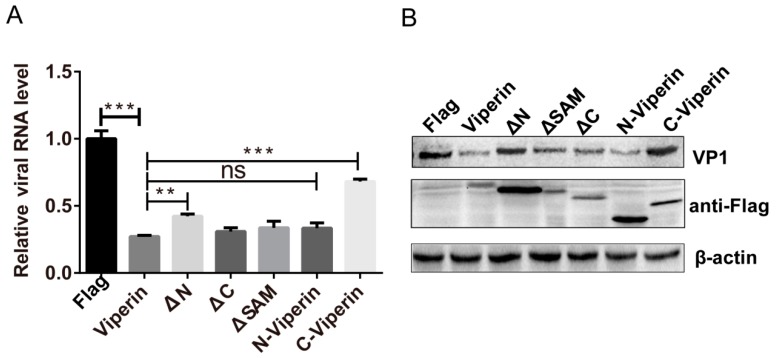
The N-terminal domain was responsible for viperin inhibition of EVA71 replication. 293T cells were transfected with FLAG-viperin, ΔN, ΔSAM, ΔC, FLAG-N-viperin, or FLAG-C-viperin for 24 h, and then infected with EVA71 at an MOI = 1 for 12 h. The cells were lysed and EVA71 (**A**) RNA levels were measured using q-PCR, while (**B**) VP1 protein levels in the infected cells were analyzed using western blotting. Data are representative of at least three independent experiments performed in triplicate (mean ± SD of fold change). ** indicates *p* < 0.01, *** indicates *p* < 0.001 and “NS” indicates there was no significant difference between the two groups.

Given that the N-terminal domain is required for viperin binding to 2C, we examined whether the N-terminal domain is responsible for the inhibition of EVA71 replication. We constructed various mutants of viperin FLAG-N-viperin (only including the N-domain) and FLAG-C-viperin (only including the C-domain). Viperin, N-viperin, C-viperin, ΔN, ΔSAM, and ΔC plasmids were transfected into 293T cells at 24 h post-transfection and then the cells were infected with EVA71 for 12 h. q-PCR and western blotting were used to analyze the effect of viperin mutants on EVA71 infection. Mutants with an N-terminal domain deletion (ΔN and FLAG-C-viperin) exerted minimal effects on the levels of EVA71 viral RNA (Figure 7A) and VP1 protein (Figure 7B), whereas mutants possessing the N-terminal domain (ΔSAM, ΔC, and FLAG-N-viperin) significantly suppressed EVA71 RNA and VP1 protein levels in a similar manner to the viperin expression plasmid. Therefore, the viperin N-terminal domain is required for EVA71 inhibition. Taken together, our results indicate that the viperin N-terminal domain of viperin was responsible for binding to 2C and inhibiting EVA71 replication, suggesting that the interaction between 2C and viperin was the mechanism behind viperin inhibition of EVA71 infection.

## 4. Discussion

In this study, we showed that viperin was induced by EVA71 infection both in vitro an in vivo and that viperin inhibited EVA71 replication. Furthermore, the 2C protein of EVA71 was found to interact with viperin through its N-terminal domain, which was also the key domain responsible for the antiviral function of viperin. Our findings suggest that viperin suppressed EVA71 infection via interacting with 2C through the viperin N-terminal domain. To the best of our knowledge, our study is the first to report this interaction in human enteroviruses.

Activation of the innate immune system following RNA viral infection results in the production of type I IFNs and the production of hundreds of ISGs [30,31]. IFNs were shown to protect both mice and cell lines against EVA71 infection [32,33]. As an important ISG, viperin has been reported to exhibit antiviral function against various viruses [34]. Accordingly, viperin is induced and upregulated via EVA71 infection and displays antiviral activity. Our results confirmed that EVA71 infection in cell lines and mice significantly induced viperin. Yogarajah et al found that viperin expression in infected SK-N-SH cells for three EV-A71 strains was significantly elevated before 24 h post infection [35]. While at 48 and 72 hpi, levels of expression were significantly reduced. This may be caused by antagonism of Type I IFN signal pathway by virus. It is known that viperin can be induced by either IFN or IFN-independent signaling pathways and can also be directly stimulated by IRF3 or IRF1 [36,37,38,39]. We found that EVA71 could upregulate viperin expression in IFNAR1 knockout mice; therefore, other IFN-independent signaling pathways may stimulate viperin upregulation. Further studies on the signaling pathways activating viperin expression are necessary.

Viperin has been demonstrated to inhibit a broad range of viruses from both DNA and RNA viral families, yet the specific antiviral mechanisms of viperin in different viral infections are diverse [12]. The antiviral function can be attributed to viperin’s interaction with cellular host factors or viral proteins. On one hand, viperin targets cellular protein for inhibition virus infection. Viperin blocks the release of influenza A virus and human immunodeficiency virus (HIV) particles by binding and inhibiting farnesyl diphosphate synthase to perturb regulation of the lipid raft on the plasma membrane. [13,14,40]. Moreover, viperin suppresses tick-borne encephalitis virus (TBEV) infection by interacting and inhibiting the function of the cellular protein Golgi Brefeldin A resistant guanine nucleotide exchange factor 1 (GBF1) to induce viral capsid particle release [24]. Also, in TBEV infection, viperin requires CIAO1 for [4Fe–4S] cluster assembly to selectively blocks positive-sense RNA amplification during the early stages of infection [41]. More importantly, viperin converses cytidine triphosphate (CTP) to ddhCTP, a chain terminator for different flavivirus RdRps [25]. On the other hand, viperin interacts with viral proteins to disrupt viral infection. Viperin suppresses HCV replication through interacting with HCV NS5A and host protein VAP-A, thus preventing the formation of replication complexes [29]. Moreover, viperin inhibits ZIKA virus and TBEV by interacting with NS3 and induce proteasome-dependent degradation of NS3 [23]. Similarly, our findings showed that viperin interacts with the 2C protein of EVA71, a protein with helicase and NTPase activities and that have an essential role in viral RNA replication [8]. We speculated that viperin affects assembly and function of the EVA71 RC by binding to the viral NTPase 2C protein. However, currently the information on how viperin function in enterovirus infection is limited. Our finding may provide some clues for viperin function in enterovirus infection.

Structurally, viperin is composed of three distinct domains, an N-terminal domain, a highly conserved central domain containing a radical SAM domain, and a C-terminal domain [42]. The specific viperin regions necessary for antiviral function between various viruses differ as does the biological effect of viperin during diverse viral infections. The C-terminal domain of viperin has been proven as the critical domain for suppressing DENV and HCV infection [15,16]. On the other hand, the SAM domain, which is predicted to function as a radical SAM enzyme [43,44], plays a key role in inhibiting HIV viral egress [14] and Bunyamwera virus replication [45]. However, the relationship between the enzyme activity of the four SAM domains and viral inhibition effects has not yet been elucidated. Recently, viperin was reported to inhibit TBEV RNA synthesis through an enzymatic Fe-S cluster and a SAM-dependent mechanism [41]. Moreover, chikungunya virus replication was found to be restricted through the viperin amphipathic helix within the N-terminal domain [22]. Our results indicated that neither the C-terminal domain nor the SAM domain were responsible for viperin inhibition of EVA71, whereas the N-terminal domain was required for virus suppression. Overexpression of the viperin N-terminal domain exhibited the same effects on EVA71 as full-length viperin. Meanwhile, deletion of the N-terminal domain not only abolishes the interaction with 2C but also EVA71 replication suppression. These findings indicated that the N-terminal domain is a critical domain for the inhibitory effects of viperin on EVA71 and the interaction between 2C and viperin. It has been reported that the N-terminal amphipathic helix is required for ER localization and interferes with the secretion of soluble proteins [28]. Viperin may inhibit the trafficking of viral and host proteins necessary for EVA71 replication through its N-terminal amphipathic helix. Furthermore, plus-stranded RNA viruses including EVA71 is considered to use membranes derived from the ER for viral replication, budding, and exit via the secretory route [46]. Furthermore, the ER is an essential organelle for enteroviruses in building replication factories and assembling the RC [47]. Thus, ER localization of viperin mediated by N-terminal domain may prevent or alter the formation of these membranous complexes, thus suppressing viral replication.

At present, the effect of viperin protein on enteroviruses is unknown. As a human enterovirus, the siRNA-mediated knockdown of viperin increases rhinovirus replication in infected epithelial cells [48]. In our study, viperin overexpression significantly suppressed EVA71 infection while viperin knockdown by siRNA enhanced EVA71 infection. Our results suggest that viperin was stimulated to suppress EVA71 infection. This phenomenon may be explained by type I IFN suppressing EVA71 replication in vivo and in vitro, as viperin (an ISG) can be stimulated by IFN and antiviral agents against EVA71 infections.

Overall, we demonstrated that viperin can be induced during EVA71 infection both in vitro and in vivo and that viperin suppressed EVA71 infection significantly. Furthermore, we found that the 2C protein of EVA71 interacts with viperin. The mechanism behind viperin suppression of EVA71 infection depends on this interaction between 2C and the N-terminal domain of viperin, which interferes with the activity of the EVA71 RC. Our findings may be helpful for the future development of new antiviral strategies that utilize viperin.

## Figures and Tables

**Table 1:** Primer and siRNA sequences used in the study.

Primers	Sequence (5′–3′)	Purpose
qPCR-mouse Viperin-F	CTATCTCCTGCGACAGCT	Real time q-PCR for mouse *viperin*
qPCR-mouse Viperin-R	AAAGCCACCTTGTAATCC	
qPCR-Viperin-F	CAAGACCGGGGAGAATACCTG	Real time q-PCR for human *viperin*
qPCR-Viperin-R	GCGAGAATGTCCAAATACTCACC	
qPCR-EVA71-VP1-F:	GAGAGTTCTATA GGGGACAGT	Real time q-PCR for EVA71 viral RNA
qPCR-EVA71-VP1-R	AGCTGTGCTATG TGA ATTAGG AA	
∆SAM 1+2- R1:	GTTGATCTTCTCCATGCGAGTGAAGTGATA	Construction of ∆SAM 1+2
∆SAM 1+2- F2:	TATCACTTCACTCGCATGGAGAAGATCAAC	
∆SAM 3+4- R1:	ATTGACTTCCTCGTCGCTGGGCAGCCGCAA	Construction of ∆SAM 3+4
∆SAM 3+4- F2:	TTGCGGCTGCCCAGCGACGAGGAAGTCAAT	
∆N- F:	ACTGCGGCCGCAGCCACCATGGATTACAAGGATGACGACGATAAGATGACCCCAACCAGCGTC	Construction of ∆N
∆C- R:	CTAGCTCGAGGCTGTCACAGGAG	Construction of ∆C
∆SAM- R1:	GACTTCCTCGTCAAAGGTGGGCAGAGGAGG	Construction of ∆SAM
∆SAM- F2:	CCTCCTCTGCCCACCTTTGACGAGGAAGTC	
C-viperein- F:	ACTGCGGCCGCAGCCACCATGGATTACAAGGATGACGACGATAAGATGTTTGACGAGGAAGTC	Construction of C-viperin
N-viperin- R:	GGTGGGCAGAGGAGGGTCCTCTT	Construction of N-viperin
∆N-42- F:	ACTGCGGCCGCAGCCACCATGGATTACAAGGATGACGACGATAAGATGGCTACCAAGAGGAGA	Construction of ∆N-42
∆N-50- F:	ACTGCGGCCGCAGCCACCATGGATTACAAGGATGACGACGATAAGATGCTGGTCCTGAGAGGG	Construction of ∆N-50
∆N-60- F:	ACTGCGGCCGCAGCCACCATGGATTACAAGGATGACGACGATAAGATGGAGGAGGAAGAGGAC	Construction of ∆N-60
∆N-65- F:	ACTGCGGCCGCAGCCACCATGGATTACAAGGATGACGACGATAAGATGCCTCCTCTGCCCACC	Construction of ∆N-65
Si-Viperin 1	GAGAATACCTGGGCAAGTT	Knock-down of *viperin*
Si-Viperin 2	TAGAGTCGCTTTCAAGATA	Knock-down of *viperin*
Si-Viperin 3	GGAGTAAGGCTGATCTGAA	Knock-down of *viperin*
